# Pharmacokinetics of recombinant human annexin A5 (SY-005) in patients with severe COVID-19

**DOI:** 10.3389/fphar.2023.1299613

**Published:** 2024-01-10

**Authors:** Brent J. Tschirhart, Xiangru Lu, Aristide Laurel Mokale Kognou, Claudio M. Martin, Marat Slessarev, Douglas D. Fraser, Aleksandra Leligdowicz, Bradley Urquhart, Qingping Feng

**Affiliations:** ^1^ Department of Physiology and Pharmacology, Schulich School of Dentistry and Medicine, Western University, London, ON, Canada; ^2^ Lawson Health Research Institute, London Health Sciences Centre, London, ON, Canada; ^3^ Division of Critical Care, Department of Medicine, Schulich School of Dentistry and Medicine, Western University, London, ON, Canada; ^4^ Robarts Research Institute, Schulich School of Dentistry and Medicine, Western University, London, ON, Canada; ^5^ Department of Microbiology and Immunology, Schulich School of Dentistry and Medicine, Western University, London, ON, Canada

**Keywords:** COVID-19, sepsis, annexin A5, SY-005, pharmacokinetics

## Abstract

**Objective:** Annexin A5 is a phosphatidylserine binding protein with anti-inflammatory, anticoagulant and anti-apoptotic properties. Preclinical studies have shown that annexin A5 inhibits pro-inflammatory responses and improves organ function and survival in rodent models of sepsis. This clinical trial aimed to evaluate the pharmacokinetic (PK) properties of the recombinant human annexin A5 (SY-005) in severe COVID-19.

**Methods:** This was a pilot randomized, double-blind, placebo-controlled trial. Severe COVID-19 patients were randomly assigned to receive intravenous 50 μg/kg (low dose, *n* = 3), 100 μg/kg (high dose, *n* = 5) of SY-005 or placebo (*n* = 5) every 12 h for 7 days. Plasma SY-005 levels were assessed using enzyme-linked immunosorbent assay (ELISA) and the PK parameters were determined using non-compartmental analysis.

**Results:** All patients treated with SY-005 had a normal baseline estimated glomerular filtration rate (eGFR, 104–125 mL/min/1.73 m^2^). Both low and high doses of SY-005 were cleared within 6 h after intravenous administration. Plasma maximum concentrations (C_max_), half-life, clearance and volume distribution of low and high doses of SY-005 were 402.4 and 848.9 ng/mL, 0.92 and 0.96 h, 7.52 and 15.19 L/h, and 9.98 and 20.79 L, respectively. Daily pre-dose circulating annexin A5 levels were not significantly different when SY-005 was administered at the low or the high dose 12-h intervals. There was no significant effect on activated partial thromboplastin time (aPTT) or INR (international normalized ratio of prothrombin time) during 7 days of SY-005 treatment.

**Conclusion:** SY-005 doses of 50 and 100 μg/kg were detectable and subsequently cleared from the plasma in severe COVID-19 patients with normal baseline renal function. There was no significant plasma SY-005 accumulation 6 h after drug administration and coagulation was not altered during 7 days of treatment.

**Clinical trials Registration:** This study was registered with ClinicalTrials.gov (NCT04748757, first posted on 10 February 2021).

## 1 Introduction

SARS-CoV-2 is a virus of the genus Betacoronavirus and causes coronavirus disease 2019 (COVID-19) (Wiersinga et al., 2020). Severe COVID-19 is complicated by multiorgan dysfunction, including acute respiratory distress syndrome (ARDS), hemodynamic instability, and coagulopathy (Matthay et al., 2020). Morbidity and mortality increase dramatically when COVID-19 is complicated by organ dysfunction (Zhou et al., 2020). To date, there have been approximately 7 million COVID-19-related deaths globally (https://covid19.who.int). Widespread mass vaccination has markedly reduced hospitalization and severity of COVID-19 (Paul et al., 2023). However, severe COVID-19 remains a clinical challenge despite available non-specific treatment options such as dexamethasone, IL-6 receptor and JAK inhibitors (Alhazzani et al., 2020; Petrelli et al., 2021; Wagner et al., 2022).

Annexin A5 is a ubiquitously expressed protein (∼36 kDa) and part of a 12-member annexin protein family (Gerke and Moss, 2002; Mui et al., 2021). Annexin A5 reversibly binds to negatively charged phospholipids, most notably phosphatidylserine, in a Ca^2+^-dependent manner. Annexin A5 has anticoagulant, anti-apoptotic and anti-inflammatory properties (Munoz et al., 2007; Ewing et al., 2011; Mui et al., 2021) and is well known as a marker to identify apoptotic cells. Annexin A5 has been shown to bind to bacteria and lipopolysaccharide (LPS) and attenuate LPS-induced tumor necrosis factor alpha (TNF-α) production (Rand et al., 2012). We recently demonstrated that annexin A5 inhibits endothelial inflammation induced by activated platelets and microvesicles in septic conditions via phosphatidylserine binding (Tschirhart et al., 2023). The first study that revealed the anti-inflammatory effects of annexin A5 in sepsis was in mice with endotoxemia in which treatment with recombinant human annexin A5 inhibited the expression of pro-inflammatory cytokines including tumor necrosis factor-alpha (TNFα) and interleukin-1β, and improved cardiac function and animal survival (Arnold et al., 2014). The findings were supported by a later study using a cecal content injection model of sepsis (Park et al., 2016). The protective effects of annexin A5 in the sepsis models are mediated in part by inhibiting LPS and high-mobility group box-1 (HMGB1) binding to the TLR4/MD2 complex (Arnold et al., 2014; Park et al., 2016). Additionally, administration of recombinant annexin A5 has been shown to inhibit thrombin formation in abdominal sepsis induced by cecal ligation and puncture in mice (Wang et al., 2018). Since severe COVID-19 is a manifestation of sepsis and involves these pro-inflammatory and coagulation pathways, annexin A5 is a viable treatment candidate (Singer et al., 2016; Mui et al., 2021).

Recombinant human annexin A5 (SY-005) has been administered to healthy subjects at intravenous doses of 0.75–20 mg per person for up to 7 days and was found to be safe (NCT04217629) (Suzhou Yabao Pharmaceutical R&D Co., Ltd, 2023). We performed a randomized, placebo-controlled pilot trial (NCT04748757) and showed that administration of SY-005 in patients with severe COVID-19 was feasible and safe (Martin et al., 2023). However, the pharmacokinetic (PK) properties of SY-005 in severe COVID-19 are unknown. In this study, we evaluated the PK profile of SY-005 administered intravenously at 50 and 100 μg/kg every 12 h for 7 days to patients with severe COVID-19.

## 2 Methods

### 2.1 Ethics

This study was approved by the Health Sciences Research Ethics Board (HSREB) at Western University and included severe COVID-19 patients from two intensive care unit (ICU) sites at Victoria Hospital and University Hospital, London Health Sciences Centre, London, Ontario. Informed consent was provided for all participants by a substitute decision maker as per the recruitment process approved by the HSREB. This study was registered under the clinical trial number NCT04748757 (Martin et al., 2023).

### 2.2 Study design

This was a pilot randomized, double-blind, placebo-controlled trial studying two doses of recombinant human annexin A5 (SY-005) in severe COVID-19 patients (Martin et al., 2023). SY-005 was produced by Suzhou Yabao Pharmaceutical R&D Co., Ltd, China, and its use as an investigational new trial drug was approved by Health Canada (Control #248696). The inclusion criteria included age >19 years, a positive polymerase chain reaction (PCR) test for SAR-CoV-2 viral infection and being admitted to the ICU for advanced monitoring and interventions. The exclusion criteria included an allergy to any component of the investigational product (annexin A5, sorbitol, or polysorbate 80), pregnancy, not expected to survive beyond 24 h, a known or suspected risk of serious bleeding complications, full dose therapeutic anticoagulation and/or acute or chronic kidney disease. SY-005 or saline was administered intravenously every 12 h for 7 days.

### 2.3 Randomization procedures

Participants were randomized (1:1:1) to a study arm (high dose treatment, low dose treatment or placebo) with randomly permuted blocks of size 3 and 6, resulting in a 2:1 ratio of participants given SY-005 compared to the placebo. Randomization was stratified by age at enrolment (65 and under or greater than 65) and by ICU site. Randomization was performed by the study pharmacist or delegate using research electronic data capture (REDCap) (Harris et al., 2009; Harris et al., 2019). All other study personnel and participants were blinded to the allocation (Martin et al., 2023).

### 2.4 Dosage preparation and administration

SY-005 was diluted based on the patient body mass and administered at a dose of 50 or 100 μg/kg body weight in 50 mL of normal saline, which could be stored at 2-8°C for up to 24 h. Doses were infused over 30 min through a dedicated venous line that was flushed with normal saline before and after administration. Placebo-treated patients received 50 mL of normal saline with no additives. SY-005 and placebo were prepared using aseptic technique (Martin et al., 2023).

### 2.5 Blood collection

Blood was obtained from an existing arterial or venous line or by venipuncture. Samples were drawn into a syringe first and then transferred into sodium citrate vacuum tubes. Blood samples were immediately placed on ice and processed within 30 min of blood collection. Blood samples were obtained pre-dose, immediately after the completion of SY-005 infusion (0 min), 15, 30 min, 1 and 6 h post-infusion. Baseline bloodwork included lab blood tests, creatinine, activated partial thromboplastin time (aPTT) and international normalized ratio (INR) of prothrombin time. The estimated glomerular filtration rate (eGFR) was calculated using the 2021 CKD-EPI equation without race (Inker et al., 2021).

### 2.6 Blood processing and plasma isolation

Whole blood was transported in a metal dewar filled with ice to a biosafety level 2^+^ laboratory. Samples were centrifuged at 1500 g for 15 min at 4°C. Plasma was removed and stored in 250 μL aliquots in a cryogenic freezing vial (Fisherbrand, Cat # 12-567-501) at −80°C.

### 2.7 Annexin A5 ELISA

To comply with the biosafety standards of Western University, frozen plasma samples were thawed and heat-inactivated at 56°C for 30 min before they were transported on ice from London Health Sciences Centre to Western University for analysis. Plasma samples were analyzed using a human annexin A5 ELISA kit (AB223863, Lot No. 2101027017, Abcam, Waltham, MA, United States). The standard curve was constructed using normal human plasma with annexin A5 levels below 0.1 ng/mL. All plasma samples were heated at 60°C for 30 min to denature annexin A5 for optimal antibody binding before they were added to the microplate wells. Colorimetric densities were obtained using a SpectraMax M5 plate reader (Molecular Devices, San Jose, CA, United States). The interday accuracy and coefficient of variation (CV) assessed at 500 ng/mL were 11.7% and 5.6%, respectively. The detection limit was 0.1 ng/mL. Since the anti-annexin A5 antibody recognizes both endogenous and recombinant annexin A5, the average endogenous annexin A5 values were subtracted from the total annexin A5 measurements to obtain SY-005 plasma concentrations.

### 2.8 Activated partial thromboplastin time (aPTT) and INR of prothrombin time

The aPTT test was measured in plasma samples that were not heat inactivated. Fifty microliters of aPTT reagent (Pacific Hemostasis aPTT-XL, Thermo Scientific, United States) were incubated with 50 μL of plasma for 5 min at 37°C in sterile 96-well plates. Clotting was initiated by adding 50 μL of CaCl_2_ (20 mM). Absorbances were determined before and after CaCl_2_ addition at 3 s intervals for 10 min at 405 nm using a SpectraMax M5 plate reader (Molecular Devices, San Jose, CA, United States) (Pratt and Monroe, 1992). To assess the INR of prothrombin time, plasma samples were incubated with the Dade^®^ Inovin^®^ reagent that contains recombinant human tissue factor, synthetic phospholipids, Ca^2+^, buffers and stabilizers to trigger coagulation. The time to the formation of a fibrin clot was measured by the transmitted light intensity using a coagulation analyzer (Sysmex CS-2500 System, Siemens, Germany).

### 2.9 Pharmacokinetic and statistical analysis

The following PK parameters were calculated with non-compartmental analysis using a software program (PCModFit V7.7, GD Allen): area under the concentration-time curve (AUC) from 0 to the last observation time (AUC_t_) and from 0 to infinity (AUC_∞_), maximum drug concentration (C_max_), terminal half-life (t_1/2_), volume distribution (Vd) and clearance (Cl) (Allen, 1990). Mann-Whitney U test was used to compare parameters between low and high-dose groups. One-way ANOVA followed by the Kruskal-Wallis test was used to compare baseline parameters among three treatment groups. A *P*-value of less than 0.05 was considered statistically significant. Statistical analysis was performed using GraphPad Prism software (version 9.5).

## 3 Results

### 3.1 Participant characteristics

A total of 17 patients were enrolled in the study, 13 of whom had biological samples available for this study in the placebo (*n* = 5), low (50 μg/kg, *n* = 3) and high (100 μg/kg, *n* = 5) dose SY-005 treatment groups. The low-dose group had only 3 patients because the consent was revoked in one patient and another patient was diagnosed with pulmonary embolism requiring therapeutic anticoagulation after randomization and before study drug administration, excluding them from the study. The PK blood samples of 4 patients were not obtained due to the unavailability of research staff on weekends and holidays. Table 1 summarizes the demographic characteristics of participants. Age, body weight, body mass index, serum creatinine, eGFR, serum albumin, platelet counts and INR were similar among the treatment groups (*p* > 0.05).

**TABLE 1:** Demographics of study patients with severe COVID-19 who were randomized to placebo, low (50 μg/kg) and high (100 μg/kg) dose SY-005 treatment groups.

Characteristics	Placebo	SY-005 (50 μg/kg)	SY-005 (100 μg/kg)	*P*-value
Number of patients (n)	5	3	5	
Age, years	54 (42–71)	37 (36–52)	46 (42–58)	0.238
Sex, male/female	4/1	1/2	5/0	0.094
Body weight, kg	104.5 (82.0–150.0)	85.0 (76.2–90.0)	93.5 (66.1–137.3)	0.170
Body mass index, kg/m^2^	34.1 (30.7–40.6)	29.4 (25.4–38.4)	29.5 (28.3–41.0)	0.539
Serum creatinine, μmol/L	78 (39–96)	46 (44–76)	57 (49–77)	0.357
eGFR, mL/min/1.73m^2^	100.2 (84.3–123.7)	122.6 (104.2–122.6)	114.6 (104.4–125)	0.237
Serum albumin, g/L	30 (27–31)	28 (27–34)	30 (25–34)	0.923
Platelet count (x10^3^/μL)	270 (215–450)	220 (216–345)	225 (132–431)	0.780
International normalized ratio (INR)	1.1 (1.0–1.3)	1.0 (1.0–1.2)	1.1 (1.0–1.1)	0.710
Endogenous annexin A5 (ng/mL)	2.04 (2.00–3.05)	1.93 (1.75–2.9)	1.99 (1.58–2.61)	0.936

Data are given as median with range in brackets. Chi-square analysis for sex and one-way ANOVA followed by the Kruskal-Wallis test for all other parameters showed no statistical significance among 3 groups. Endogenous annexin A5 plasma levels were determined before SY-005 or placebo treatment. eGFR, estimated glomerular filtration rate.

### 3.2 Pharmacokinetics of SY-005

**FIGURE 1:**
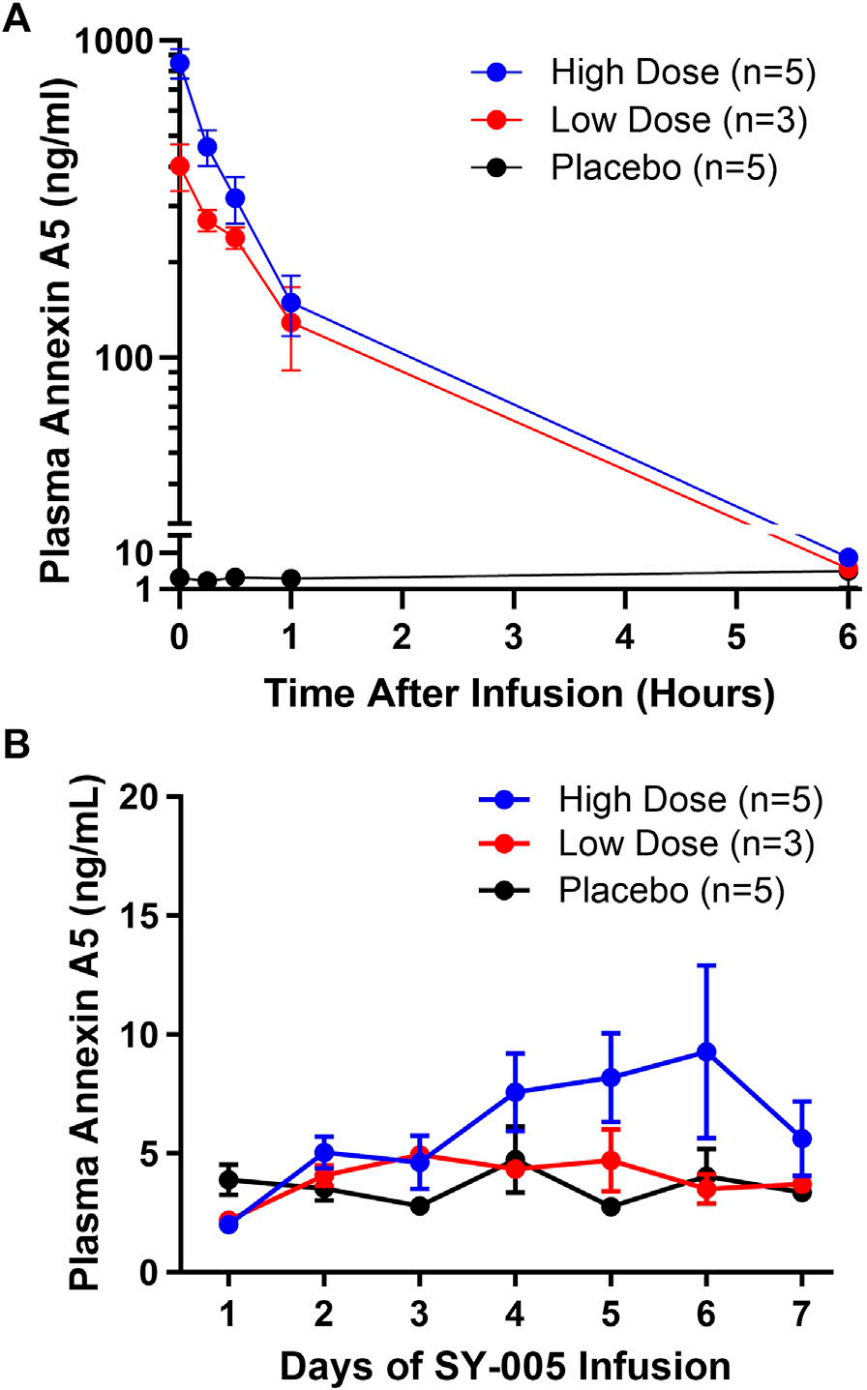
Pharmacokinetics of SY-005 and daily pre-dose plasma annexin A5 levels in patients with severe COVID-19. **(A)** Study day 1 plasma annexin A5 concentrations at the end of the 30-min infusion of placebo (saline), low (50 μg/kg) or high (100 μg/kg) dose SY-005 intravenous infusion (time 0 h) and at 15 min, 30 min, 1 h, and 6 h. **(B)** Evolution of daily pre-dose plasma annexin A5 levels during SY-005 infusion q12h for 7 days.

Endogenous annexin A5 levels before SY-005 administration were similar among the three treatment groups (Table 1). Placebo treatment did not change plasma annexin A5 levels (Figure 1A). SY-005 treatment dose-dependently increased plasma SY-005 levels with C_max_ of 402.4 and 848.9 ng/mL, which decreased to 3.7 and 7.4 ng/mL at 6 h following 30 min of 50 and 100 μg/kg infusion, respectively (Figure 1A; Table 2). C_max_ was significantly higher in the high dose than in the low dose SY-005 treatment group (*p* < 0.05) and was increased by 201- and 424-fold over endogenous annexin A5 levels in low and high dose groups, respectively. The terminal phase elimination half-life (t_1/2_) of SY-005 was similar between low and high dose groups (0.92 vs. 0.96 h). All other pharmacokinetic parameters including λz, AUC_t_, AUC_∞_, clearance, mean residence time (MRT), volume distribution (Vd) and volume distribution at steady state (Vss) were not significantly different between low and high-dose SY-005 treatment groups (*p* > 0.05, Table 2).

**TABLE 2:** Pharmacokinetics of SY-005 following a 30-min intravenous infusion in severe COVID-19 patients.

Parameters	SY-005 (50 μg/kg)	SY-005 (100 μg/kg)	*P-*value
Number of patients (n)	3	5	-
T_max_(h)	0	0	-
C_max_(ng/mL)	402.4 ± 116.2	343.9 ± 200.9	0.036
AUC_t_(h*ng/mL)	404.3 ± 55.0	551.1 ± 159.4	0.250
${\text{AUC}}_{\infty }$ (h*ng/mL)	409.7 ± 54.0	562.0 ± 159.9	0.250
λ z(1/h)	0.757 ± 0.024	0.739 ± 0.121	0.736
Half-life,t_1/2_(h)	0.92 ± 0.03	0.96 ± 0.16	0.736
Clearance (L/h)	7.52 ± 1.56	15.19 ± 7.14	0.071
MKT (h)	0.37 ± 0.06	0.37 ± 0.19	1.000
Vd (L)	9.93 ± 2.33	20.79 ± 3.77	0.071
Vss (L)	6.43 ± 0.39	12.60 ± 4.33	0.071

Data are mean ± standard deviation (SD) and analyzed by Mann Whitney U test. T_max_, maximum drug time; C_max_, maximum drug concentration; AUC_t_, area under the curve until the last observation time; AUC_∞_, area under the curve extrapolated to infinity; λz, terminal phase elimination rate constant; MRT, mean residence time; Vd, volume distribution; Vss, steady state volume distribution.

Annexin A5 plasma levels were determined daily immediately prior to the morning SY-005 treatment. Neither the low nor high-dose SY-005 treatment increased daily pre-dose circulating annexin A5 levels (Figure 1B), suggesting minimal plasma SY-005 accumulation in either of the treatment groups. Baseline eGFR was normal in patients who received SY-005 (range 104.2–125.0 mL/min/1.73 m^2^) and there was no relationship between eGFR and SY-005 clearance in this study population.

### 3.3 Effects of SY-005 on coagulation

**FIGURE 2:**
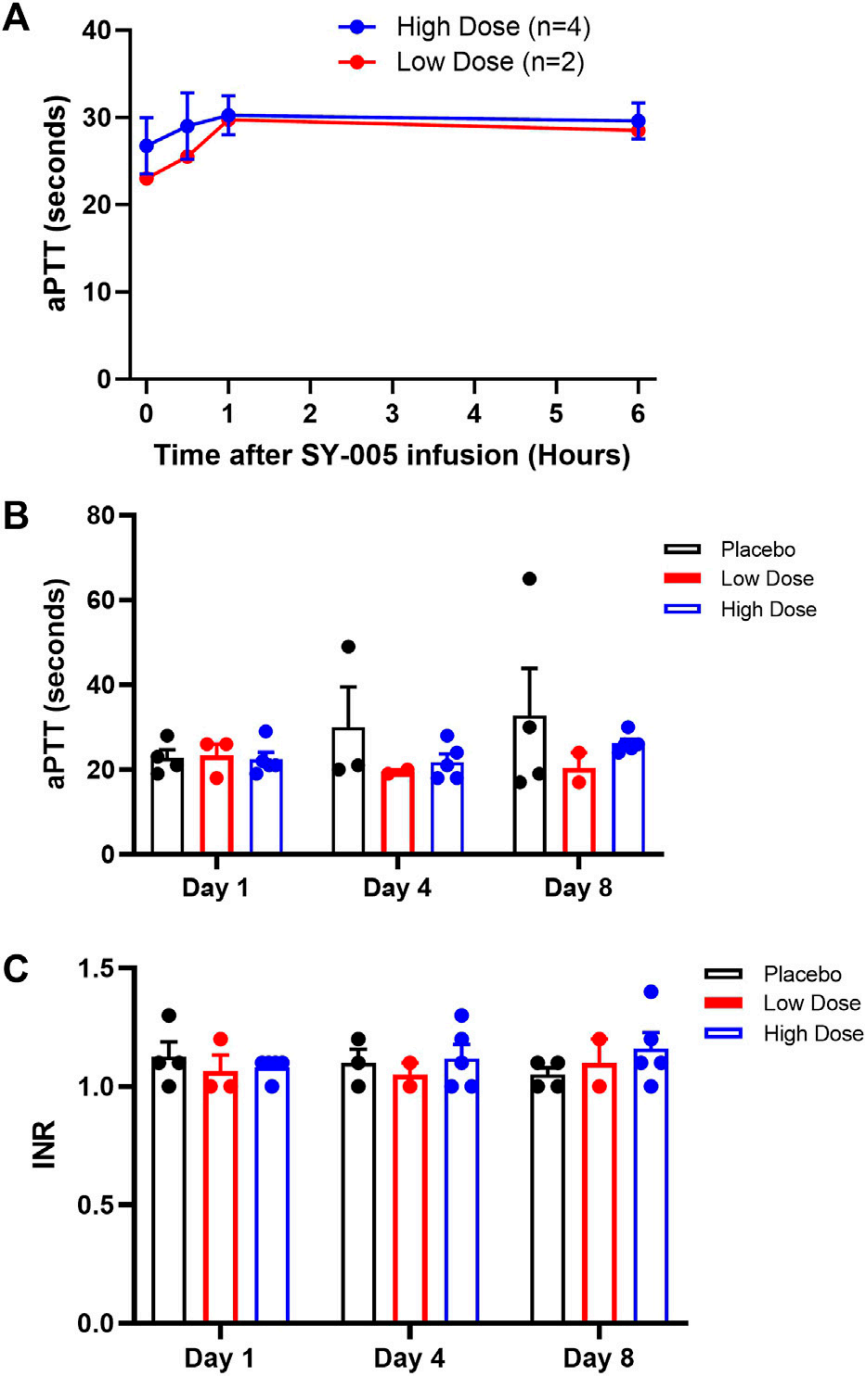
Effects of SY-005 on blood coagulation. **(A)** Activated partial thromboplastin time (aPTT) during the first 6 h of SY-005 administration on study day 1. **(B,C)** aPTT and INR measurements, respectively, on study days 1, 4, and 8 before SY-005 or placebo administration. Due to heat inactivation, one sample from each dose group was not suitable for aPTT test resulting in *n* = 4 and *n* = 2 for high and low-dose groups, respectively. Data are mean ± SEM except in A where means of low dose group are shown. There was no statistical significance in **A**, **B** or **C**.

Severe COVID-19 can be complicated by coagulopathy (Godoy et al., 2020), and annexin A5 has been shown to have a moderate anticoagulant effect (Gerke and Moss, 2002; Mui et al., 2021). To assess the effects of SY-005 on blood coagulation, aPTT was quantified on study day 1 during the first 6 h after SY-005 administration. Additionally, aPTT and INR were determined at baseline (day 1), day 4 and day 8 after SY-005 treatment. Both low and high doses of SY-005 did not significantly affect aPTT in the first 6 h (Figure 2A), and there was no significant effect on aPTT (Figure 2B) or INR (Figure 2C) at day 4 and day 8 during SY-005 treatment.

## 4 Discussion

This study examined the PK profile of two doses of SY-005 (50 and 100 μg/kg) in a randomized, double-blind, placebo-controlled pilot trial in severe COVID-19 patients with normal renal function. Our data show that the infusion of SY-005 dose-dependently increased C_max_, λz, AUC_t_ and AUC_∞_. SY-005 was eliminated rapidly from the plasma with a clearance of 7.52 and 15.19 L/h and an elimination half-life of 0.92 and 0.96 h for low and high doses, respectively, indicating a rapid elimination in patients with severe COVID-19. Notably, there were no drug-related serious adverse events in this pilot clinical trial (Martin et al., 2023). These results are important for planning further studies of this novel therapeutic agent for the treatment of COVID-19 and sepsis.

Endogenous annexin A5 in all treatment groups was similar to the normal basal circulating plasma levels of healthy volunteers (Matsuda et al., 2003; Van Heerde et al., 2003; Burgmaier et al., 2017). Plasma SY-005 concentrations were obtained by subtracting endogenous levels from total annexin A5 concentrations. Our data show that SY-005 clearance and elimination half-life are similar to that of healthy volunteers in a phase I trial of SY-005, in which this drug was cleared within 6 h post-infusion at 5 and 10 mg per person, which are equivalent to 41 and 82 μg/kg doses, respectively (NCT04217629; Suzhou Yabao Pharmaceutical R&D Co., Ltd, 2023). This PK profile supports more frequent intravenous dosing, possibly every 6–8 h to achieve adequate therapeutic effect in future trials.

Annexin A5 has been used to label apoptotic cell death for diagnostic imaging in patients with cancer (Corsten et al., 2006) and acute myocardial infarction (Hofstra et al., 2000) because of its ability to bind to the externalized phosphatidylserine, a characteristic feature of apoptosis. The ^99m^Tc-labelled annexin A5 administrated intravenously in patients with acute myocardial infarction displayed a half-life of 17–62 h with a clearance of 1.2–2.8 L/h (Boersma et al., 2003). The longer half-life and lower clearance of radiolabeled annexin A5 are attributed to the radiochemical properties of the conjugated annexin A5 with radio ligand ester or thiolate bonds (Boersma et al., 2003; Boersma et al., 2005).

Acute kidney injury occurs in 20%–40% of COVID-19 patients admitted to the ICUs, and approximately 20% of these patients require renal replacement therapy (Ronco et al., 2020; Chan et al., 2021). Renal dysfunction reduces the elimination of drugs and their metabolites that are primarily cleared by the kidneys. Several reports identified that the accumulation of radiolabelled annexin A5 in humans and rodents was most pronounced and rapid in the kidney and that 57% of the injected dose was excreted in the urine around their half-life (t_1/2_), suggesting that kidneys are the main route of elimination of the radiolabelled annexin A5 (Boersma et al., 2003; Boersma et al., 2005; Cheng et al., 2013). The metabolism of the administered recombinant human annexin A5 is currently unknown. Annexin A5 can be degraded by protease K, a broad-spectrum serine protease derived from Tritirachium album, and by lysosomal proteinases (Cuervo et al., 2000). However, the exact enzymes that degrade annexin A5 are not clear. Annexin A5 is a structurally stable protein. SY-005 has the same amino acid sequence without acetylation of its N-terminal alanine as compared to the endogenous annexin A5. Whether the recombinant human annexin A5 following intravenous infusion is degraded and/or eliminated intact by the kidneys due to its relatively small molecular size (36 kDa) remains to be investigated. In our study, all COVID-19 patients had normal baseline eGFR (104–125 mL/min/1.73 m^2^) and serum creatinine levels. Both low and high doses of SY-005 were cleared within 6 h from plasma following intravenous infusion. Further, intravenous infusions of either low or high doses of SY-005 every 12 h for 7 days did not cause significant accumulation in plasma. Future studies should investigate the impact of acute and chronic kidney disease on SY-005 clearance, pharmacokinetics, and potential complications.

Coagulopathy occurs in severe COVID-19 patients and its clinical manifestations include a hypercoagulable state, venous and arterial thrombosis, and disseminated intravascular coagulation (DIC) (Godoy et al., 2020). In the present study, patients with suspected risk of bleeding or on therapeutic anticoagulation were excluded. Baseline platelet account, aPTT and INR were normal in all patients. SY-005 treatment acutely within 6 h and with daily q12h administration for 7 days did not affect aPTT or INR, suggesting that SY-005 does not significantly alter blood coagulation in patients with severe COVID-19. The results are consistent with previous studies that annexin A5 does not prolong bleeding times in rodents even at very high doses (1.0 mg/kg body weight, i.v.) (Römisch et al., 1991).

This study has limitations. First, the sample size is small due to decreasing admissions of patients with COVID-19, which triggered a stopping rule for the trial. Second, urine samples were not collected, and urine SY-005 levels were not determined due to logistical challenges associated with clinical care for COVID-19 patients in our ICUs. The elimination of SY-005 in urine needs to be confirmed in future studies. Lastly, all patients in our study had normal kidney function. It is unknown if the clearance of SY-005 is affected by renal dysfunction.

## 5 Conclusion

In severe COVID-19, the pharmacokinetics of SY-005 dosed at 50 and 100 μg/kg in patients with normal renal function showed a dose-dependent increase in SY-005 plasma levels, with subsequent rapid clearance. SY-005 did not accumulate in plasma, and there was no significant effect on blood coagulation during the 7-day study period. These data suggest that SY-005 dosed at either 50 or 100 μg/kg achieved blood concentrations in COVID-19 patients with normal renal function that are compatible with therapeutic dose without causing harm, supporting its further investigation in a larger randomized clinical trial.

## Data Availability

The raw data supporting the conclusions of this article will be made available by the authors, without undue reservation.
